# Synthetic Circular miR-21 Sponge as Tool for Lung Cancer Treatment

**DOI:** 10.3390/ijms23062963

**Published:** 2022-03-09

**Authors:** Ana R. Rama, Francisco Quiñonero, Cristina Mesas, Consolación Melguizo, Jose Prados

**Affiliations:** 1 Institute of Biopathology and Regenerative Medicine (IBIMER), Center of Biomedical Research (CIBM), University of Granada, 18016 Granada, Spain; fjquinonero@ugr.es (F.Q.); cristinam@ugr.es (C.M.); melguizo@ugr.es (C.M.); jcprados@ugr.es (J.P.); 2 Institute of Biosanitary Research from Granada (ibs. GRANADA), 18012 Granada, Spain; 3 Department of Health Sciences, Faculty of Health Sciences, University of Jaen, 23071 Jaen, Spain; 4 Department of Anatomy and Embryology, Faculty of Medicine, University of Granada, 18016 Granada, Spain

**Keywords:** miRNA, circular sponges, miRNA-21, lung cancer

## Abstract

Lung cancer is the most common cancer in the world and several miRNAs are associated with it. MiRNA sponges are presented as tools to inhibit miRNAs. We designed a system to capture miRNAs based on circular RNAs (circRNA). To demonstrate its usefulness, we chose miR-21, which is upregulated and implicated in lung cancer. We constructed a miR-21 sponge and inserted it into a vector that facilitates circular RNA production (Circ-21) to study its effect on growth, colony formation, and migration in lung cancer cell lines and multicellular tumor spheroids (MTS). Circ-21 induced a significant and time-dependent decrease in the growth of A549 and LL2 cells, but not in L132 cells. Furthermore, A549 and LL2 cells transfected with Circ-21 showed a lower number of colonies and migration than L132. Similar findings were seen in A549 and LL2 Circ-21 MTS, which showed a significant decrease in volume growth, but not in L132 Circ-21 MTS. Based on this, the miR-21 circular sponge may suppress the processes of tumorigenesis and progression. Therefore, our system based on circular sponges seems to be effective, as a tool for the capture of other miRNAs.

## 1. Introduction

Lung cancer is the cancer that occurs most frequently in the world, and the most in men, and the third most in women in terms of mortality [1]. It has a low survival rate because the disease is generally advanced or metastatic at the time of diagnosis. Non-small cell lung cancer (NSCLC) represents 80–85% of all lung cancer diagnoses, with a five-year survival rate of 5% [2]. This aggressiveness and the need of more effective treatments have led to the study of miRNAs, which are found suppressed or overexpressed in this type of cancer.

MicroRNAs (miRNAs) are short non-coding RNAs composed of ∼22 nucleotides that induce posttranscriptional gene silencing in their target through binding to 3′ UTR mRNA [3,4]. The miRNAs are involved in diverse biological processes such as cell cycle control [5], cellular differentiation [6], development [7], and metabolism [8]. They are also involved in diseases such as diabetes [9], neurodegenerative diseases [10], and cancer [11,12]. In the last decade, deregulation in the expression of miRNAs has been related to the appearance of several cancers [13,14,15] similar to lung cancer, both with oncogenic and tumor suppressor functions (Table S1).

In addition, miRNAs show expression profiles characterized by differences between normal and tumor tissues, as well as between tumor types, which allows them to be used as biomarkers for determining prognosis, response to chemotherapy, prediction of the efficacy of treatment, and a patient’s susceptibility to disease [16]. The miR-183 cluster is a miRNA family with sequence homology comprising miRNAs-183, -96, and -182. Zhu et al. [16] showed that miR-96 and miR-183 were found at considerably high levels in the serum of people affected by NSCLC. In addition, it was found that miR-183 was overexpressed in these tumor cells, which was associated with metastasis in advanced NSCLC. miR-21, which is upregulated in several cancers (Table S2), including lung cancer, and is involved in tumorigenesis, progression, and metastasis processes [17,18,19]. High expression levels have been associated with both clinicopathological factors and prognosis of the patient and tumor, and can be used as a diagnostic and prognostic biomarker [20,21].

miRNA sponges are presented as tools to inhibit miRNAs. A miRNA sponge is an mRNA sequence of multiple tandem binding sites (MBS) to targeted specific miRNAs, which allows for inhibition of expression of diverse miRNAs of the same family [22]. A “seed” sequence determines the binding specificity of the miRNA towards the end of 3′ UTR of its mRNA target, and contains certain bases which do not pair with the mRNA target, creating a small loop. This sequence determines the binding specificity of the miRNA toward the end of the 3′ UTR of its mRNA target [4,23]. In addition, the sponges containing bulged sites, which are mispaired opposite to miRNA positions 9–12, show a better and more stable binding efficacy than sponges with complete complementarity [24]. This may be caused by an increase in endonucleolytic cleavage activity of AGO2 in the perfect binding of the miRNA that leads to the degradation of the sponge [25]. The use of circular sponges prevents this degradation.

Circular RNAs (circRNAs) are covalently closed single-stranded RNAs that lack 5′ ends and 3′ poly-A structures. Circ-RNAs are found predominantly in the cell cytoplasm, passing through the nucleus through pores, or through the rupture of the nuclear envelope during mitotic division [26]. Despite this, the mechanism of how nuclear output to the cytoplasm might be controlled is still unknown [27]. As for their synthesis, exons that form circRNAs have been observed to often be surrounded by unusually long introns in which splicing is believed to be less efficient. Furthermore, these introns in humans are enriched in ALU repeats [26], which are intronic elements that promote circularization [28]. Normal RNA splicing is formed from pre-RNA by a donor site binding upstream and an acceptor site downstream, resulting in a linear RNA transcript. However, circRNA are formed by backsplicing, a variation of this splicing mechanism. The first step of the back-splicing occurs at the point of branch upstream from the exon of circularization where the 2′-hydroxyl group attacks the 5′ splice site. In the second step, the end was previously generated in the 3′-hydroxyl from the circularizing exon attacks the 3′ splice site, resulting in the release of the circular exon that has covalently joined ends [29]. The efficiency of backsplicing will depend on the general secondary structure of the pre-mRNA transcript and thus varies with the pairing of different exon sequences and intron structures [30]. Unlike linear RNAs, circRNAs are more stable in that they form covalently closed continuous RNA loops, and, therefore, are inherent to exonucleolytic degradation of RNA [26,27,31,32,33].

The aim of this work was to demonstrate the use of circular sponges as a tool in capturing miRNA. As an example, we chose miR-21 for its role in lung cancer. We designed the circular sponge to capture miR-21 and to study the effects on cell proliferation, migration, and apoptosis in lung cancer cell lines and multicellular tumors. For this purpose, we constructed a sponge consisting of seven tandem-arrayed miR-21 binding sites, which were inserted between two ALU sequences of a special vector downstream from the CMV (cytomegalovirus) promoter to recirculate the sponge, and used *EGFP* (*enhanced green fluorescent protein*) as a reporter gene. This new system may restore upregulated miR-21 expression levels in lung cancer.

## 2. Results

### 2.1. miR-21 Differential Expression in Lung Cancer Cell Lines

**Figure 1:**
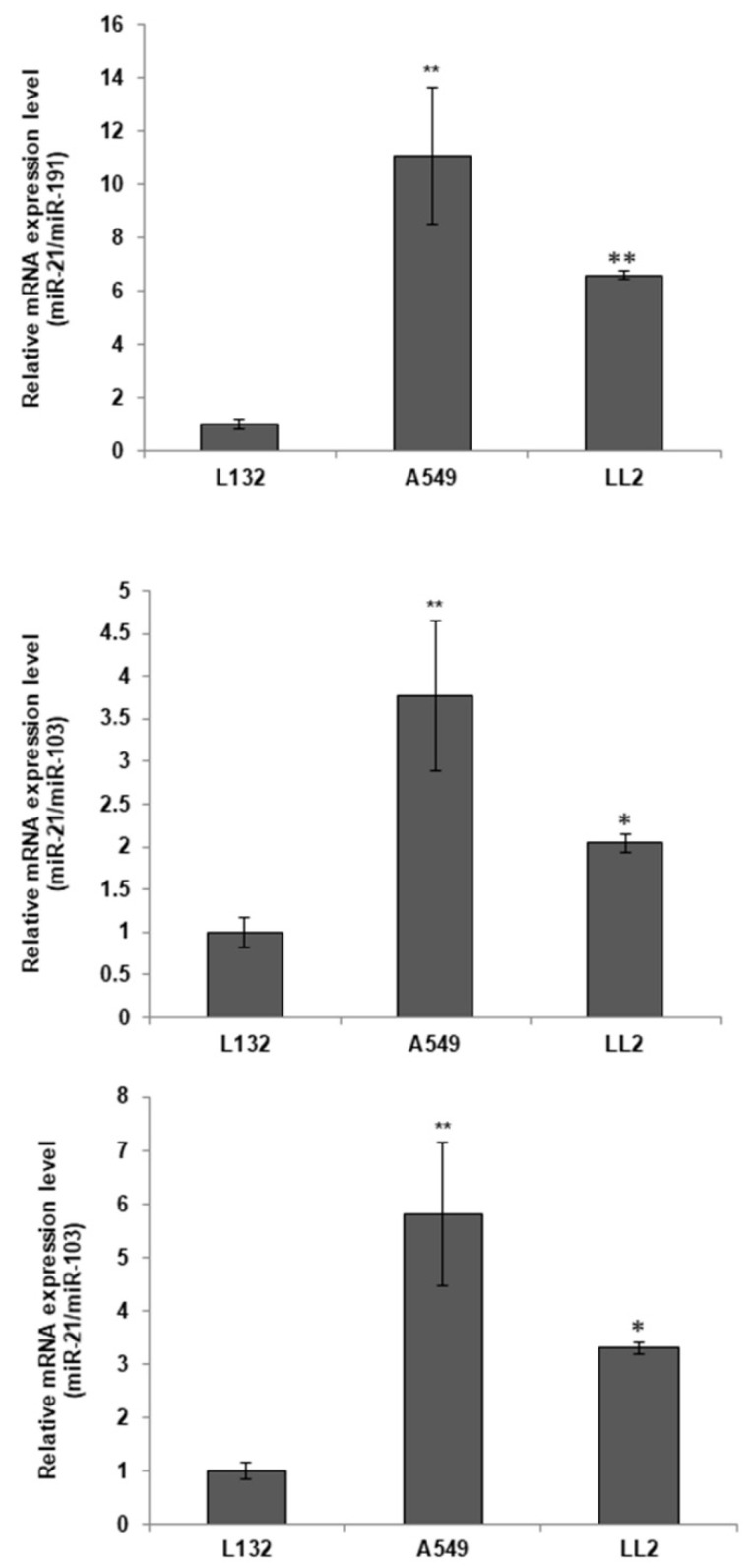
Basal expression of miR-21 in L132, A549, and LL2 and cell lines. Values represent means ± SD of triplicate cultures. Statistical analysis was performed using a two-tailed *t*-test comparing against the non-tumor line (L132). * *p* < 0.05 and ** *p* < 0.01.

A qPCR test was conducted to determine the miR-21 expression levels in the A549, LL2 and L132 lung cancer cell lines. miR-103a and miR-191 were used for housekeeping. As shown in Figure 1, the expression levels of miR-21 were higher in A549 cell line than in both LL2 and L132 lung cancer cell lines. L132 cells showed the lowest levels of miR-21 expression.

### 2.2. Development of a Simple and Effective miRNA Sponge Expression System

**Figure 2:**
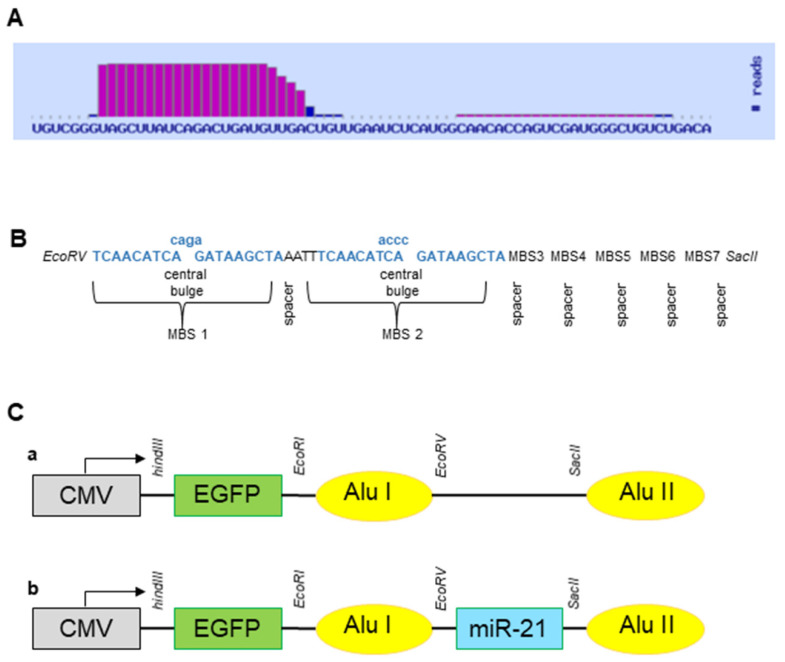
miR-21 sponge expression system. (**A**) hsa-miR-21-5p sequence from miRNABase database. (**B**) Design of the miR-21 sponge. (**C**) Sequence of subcloned into pcDNA3.1 (+) CircRNA Mini Vector: (**a**) Subcloned *EGFP* gene upstream of Alu sequence (Circ-EGFP); (**b**) miR-21 was in Circ-EGFP between Alu sequences (Circ-21).

The *EGFP* gene was subcloned into pcDNA3.1 (+) CircRNA mini vector (Addgene, Teddington, UK) (Circ-EGFP) to be used as a reporter gene. hsa-miR-21-5p was provided by the miRNABase database (Figure 2A), and the miR-21 sponge was designed according to the Section 4 described above (Figure 2B). This was synthesized (IDT, Coralville, IO, USA) and subcloned into Circ-EGFP (Circ-21) (Figure 2C). The correct sequence construct was analyzed through sequencing (Supplementary Data S1).

### 2.3. Correct Expression of Sponges In Vitro

**Figure 3:**
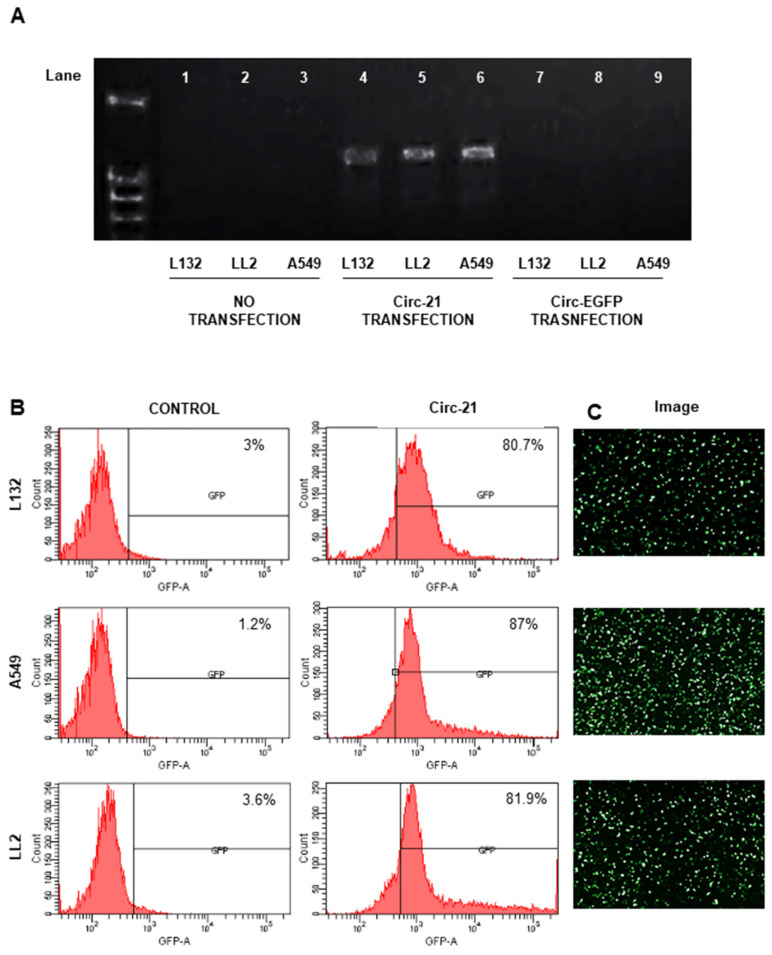
Detection of sponge miR-21 expression by PCR (32 cycles). (**A**) Lanes 1, 2 and 3, no transfected cells; Lanes 4, 5 and 6, cells transfected with Circ-21; and Lanes 7, 8 and 9, cells transfected with Circ-EGFP. (**B**) Flow cytometry analysis of EGFP expression. (**C**) EGFP fluorescence representative images (48 h after transfection) (4×).

MiR-21 sponge expression was confirmed using RT-PCR (Figure 3A). The *EGFP* gene was used as a gene reporter, which helped to discover the transfection efficiency of Circ-21 (~83%) (Figure 3B).

### 2.4. Inhibition of Cell Growth by Circ-21

**Figure 4:**
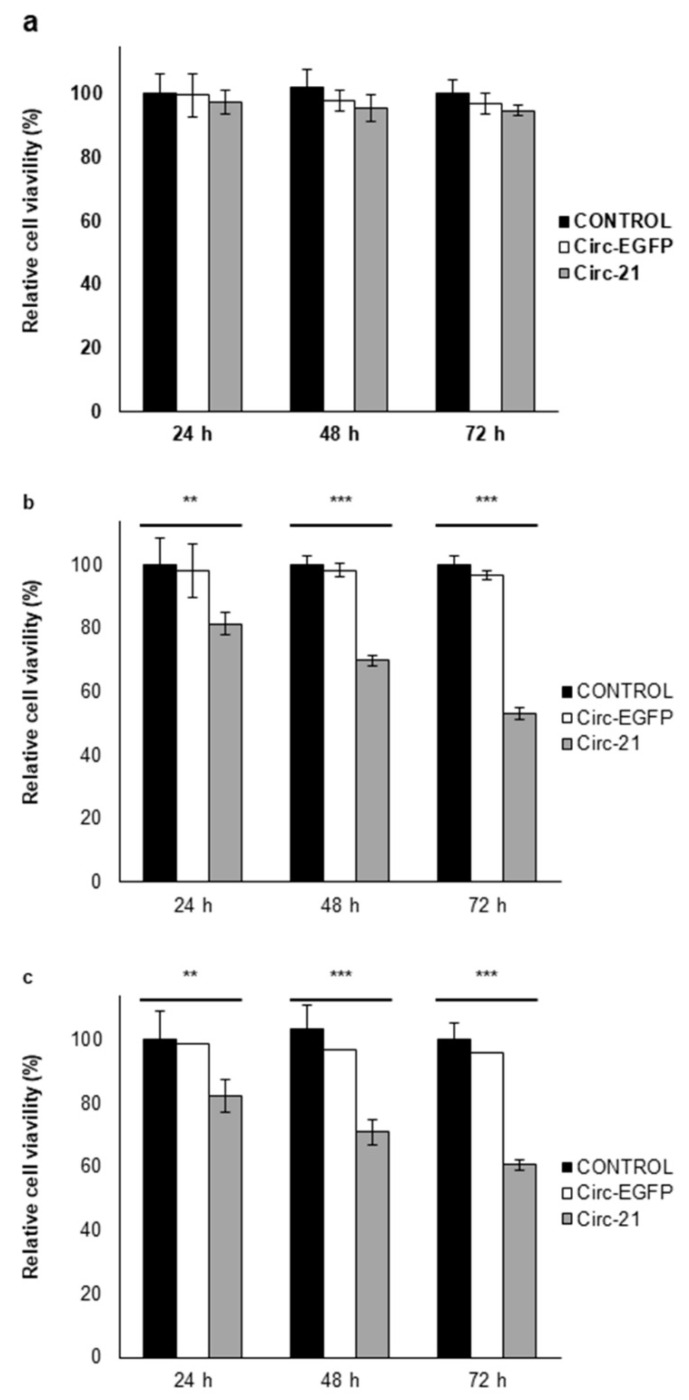
Effect of Circ-21 sponge on cell proliferation. (**a**) L132; (**b**) A549; (**c**) LL2. Values represent means ± SD of triplicate cultures. Statistical analysis was performed using a two-tailed *t*-test comparing the different samples against the control. ** *p* < 0.01 and *** *p* < 0.001. Only statistically significant differences are shown and indicated with asterisks.

As shown in Figure 4, A549 and LL2 cell lines showed a significant and time-dependent decrease in growth, reaching the maximum percentage of inhibition at 72 h (46.8% and 39.4%, respectively) (*p* < 0.001). By contrast, no difference was observed here in L132 cells (with low levels of miR-21), demonstrating the selective target of Circ-21 to lung cancer cells, with high levels of miR-21 expression, thereby causing inhibition of their cell growth. Cell lines transfected with Circ-EGFP (control) showed similar proliferation to that of each parental cell.

### 2.5. Inhibition of Cell Migration by Circ-21

**Figure 5:**
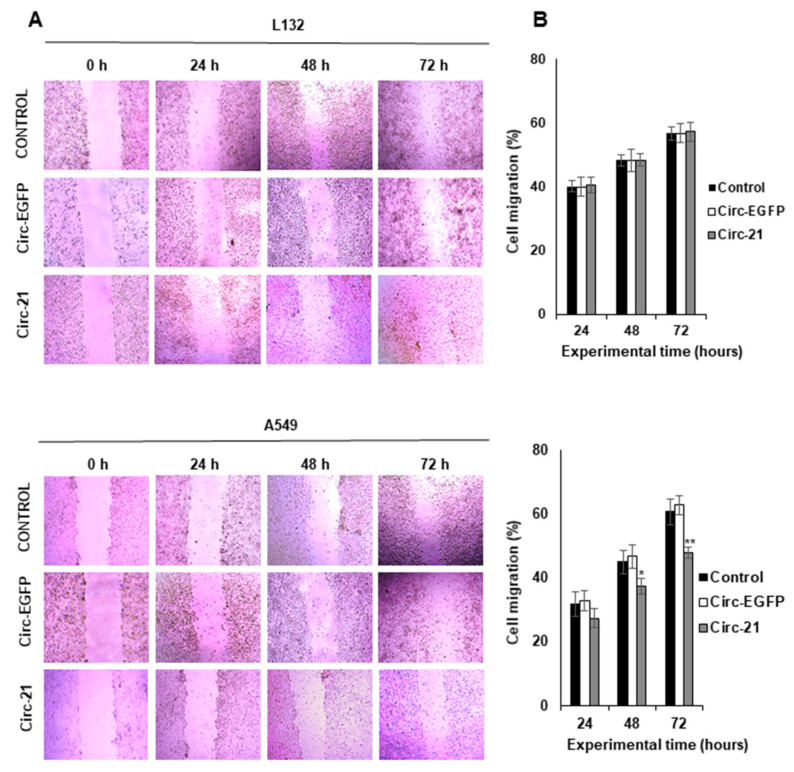
The Circ-21 sponge inhibited lung cancer cell migration. (**A**) Representative microscopy images of wound-healing assays; (**B**) Graphic representation of the percentage of lung cancer cells tumor migration (area of the scratch). Values represent means ± SD of triplicate cultures. Statistical analysis was performed using a two-tailed *t*-test comparing the different samples against the control. * *p* < 0.05 and ** *p* < 0.01. Only statistically significant differences are shown and indicated with asterisks.

As shown in Figure 5, the A549 cell line transfected with Circ-21 showed a significant decrease in tumor cell migration vs. controls (Circ-EGFP transfected cells and non-transfected cells) at 48 and 72 h of the assay. The highest reduction was detected at 72 h (12.8%; *p* < 0.01). By contrast, this reduction was not observed in the L132 cell line. The wound healing assay could not be performed on the LL2 cell line because while trying to create the wound, the monolayer culture became detached.

### 2.6. Colony Formation

**Figure 6:**
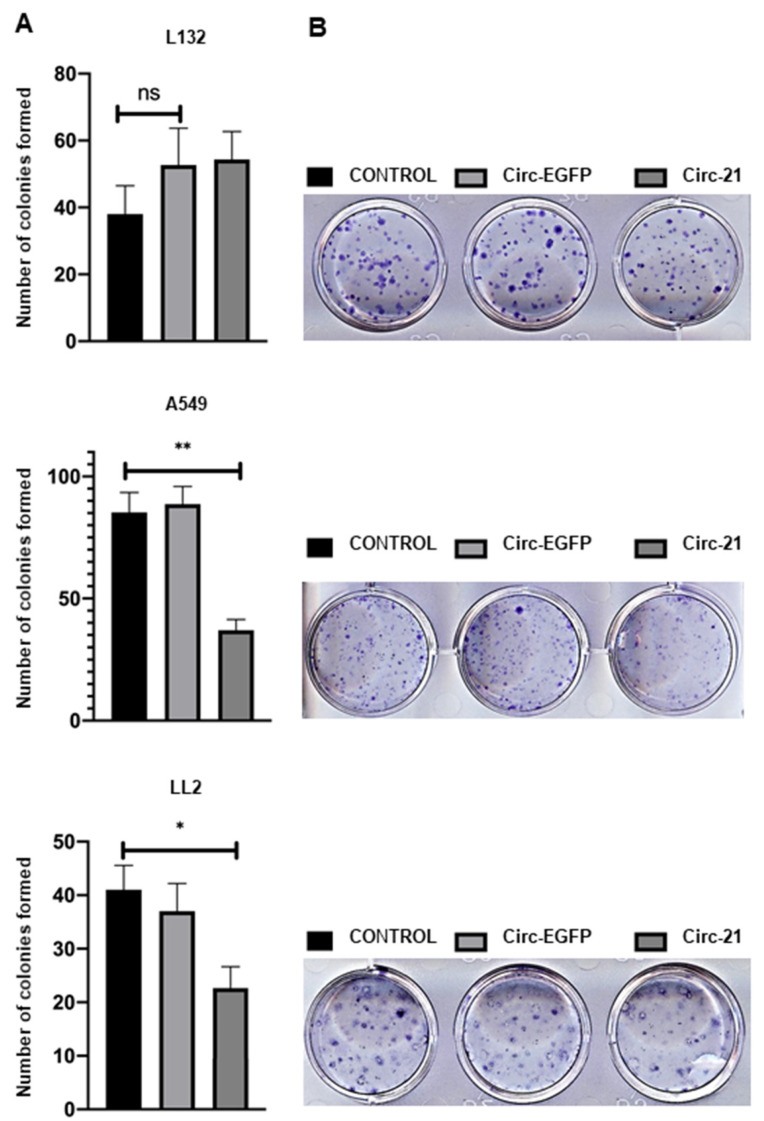
Effects of the miR-21 sponge on colony formation. (**A**) Colony formation assay. Values represent means ± SD of triplicate cultures. Statistical analysis was performed using a two-tailed *t*-test comparing the different samples against the control. * *p* < 0.05 and ** *p* < 0.01. Only statistically significant differences are shown and indicated with asterisks. ns = not statistically significant. (**B**) Representative image of wound-healing assay colony formation.

Cell colony formation exhibited a lower colony number in A549 and LL2 cells transfected with Circ-21 (*p* < 0.01 and *p* < 0.1, respectively) compared with both cell lines transfected with Circ-EGFP or no transfection (control) (Figure 6). In contrast, no differences in the number of colonies were detected in L132 cells transfected or not transfected.

### 2.7. miR-21 Sponge Therapy Effect in 3D Tumor Spheroid Models

**Figure 7:**
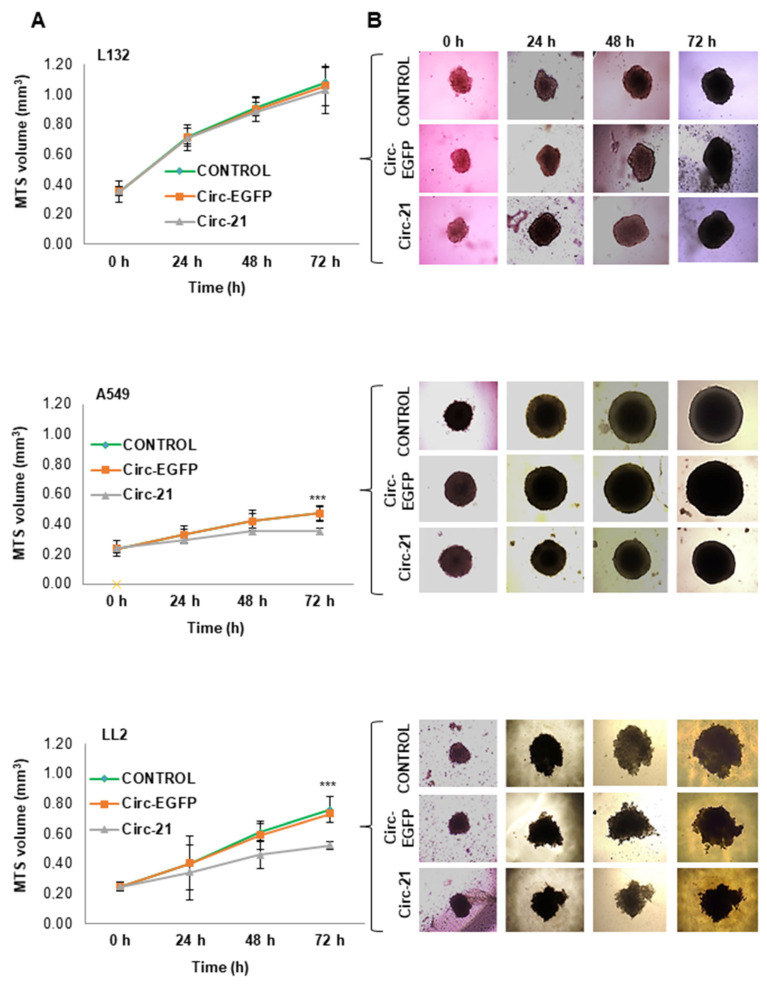
The miR-21 sponge in multicellular spheroids (MTS). (**A**) Volume growth. Values at 24, 48 and 72 h means ± SD of quintupled cultures. Statistical analysis was performed using a two-tailed *t*-test comparing the different samples against the control. *** *p* < 0.001. Only statistically significant differences are shown and indicated with asterisks. (**B**) Representative microscopy images of MTS.

As shown in Figure 7, MTS A-549 and LL2 transfected with Circ-21 exhibited a significant and time-dependent decrease in volume growth that reached a maximum at 72 h (31.2% and 25.8%, respectively) (*p* < 0.001). In contrast, minimal growth inhibition was observed in L132 (4.8% at 72 h). MTS from cells transfected with Circ-EGFP and non-transfected cells (control) showed similar behavior.

## 3. Discussion

Lung cancer exhibits a high incidence rate, a low five-year survival and a high mortality, which makes it one of the most dangerous and complicated cancers to treat [1,2]. Gene therapy is presented as one of the most promising fields in the development of the future lung cancer therapies, and within this field, miRNAs can be used as therapeutic targets [4,34]. The appearance of various cancers has been related to the dysregulation in the expression of miRNAs [13,14,15], allowing for the establishment of characteristic miRNA expression profiles that differentiate between normal and tumor tissues, as well as between tumor types. These characteristic profiles can be used as biomarkers to determine prognosis, response to chemotherapy, prediction of treatment efficacy, and susceptibility of a patient to disease, which overexpression or down expression allows. Zhao et al. [20] analyzed miR-21 expression levels in the sera of 80 NSCLC and 60 healthy people by real-time PCR, detecting higher relative serum miR-21 levels in NSCLC patients than that in healthy people. In addition, NSCLC patients with a higher relative expression had significantly lower survival times than those in the lower expression. Therefore, miR-21 may be useful as a diagnostic and prognostic indicator of NSCLC.

We studied the expression level of miR-21 in A549 human lung adenocarcinoma epithelial cell line, LL2 mouse Lewis lung carcinoma cell line, and L132 human embryonic lung cell line. has-miR-103-3p and has-miR-191-5p were used for housekeeping in qPCR analyses, revealing that the expression levels of miR-21 were higher in A549 and LL2 cell lines than in L132. Similar results were obtained by Gallach et al. [35], whose expression analysis of miR-21 by qPCR was also increased in the A549 cell lines.

Overexpression of miR-21 is related to the processes of cell proliferation, metastasis, invasion and chemoresistance in multiple types of cancer, including lung cancer [36,37]. Downregulation of miR-21 in A549 cells has displayed sensitization to radiation therapy, decreasing the ability to survive and proliferate, while the rate of cellular apoptosis continues to increase [38].

We propose the use of a circular miRNA sponge to decrease the expression levels of miR-21 in lung cancer cell lines, therefore inhibiting migration and proliferation and inducing apoptosis. mRNA sponges are a sequence of multiple tandem binding sites (MBS) of mRNA molecules able to abduct miRNA molecules [4,22], allowing for inhibiting the expression of diverse RNAs of the same family [39]. Lavenniah et al. [33] designed several sponges with spacers of different lengths (6, 12, 24, 36, and 72 nt), and with bulging or perfect complementary miRNA binding sites, to determine their efficiency in sponging the mir-132 and miR-212 targets. The 12 nt spacer sponge produced the greatest rescue effect, which decreased with larger spacers sizes. Also, this effect increased with an increasing number of binding sites, but no significant difference was seen between circRNAs containing 12 and 16 binding sites. Finally, the perfect circRNAs, carrying 12 miRNA binding sites separated by 12-nt spacers, were degraded to a much greater extent than bulged circRNAs (785-fold and 27-fold respectively). We designed an miR-21 sponge with 7 multiple tandem binding (MBS), based on hsa-miRNA-21-5P sequences (miRBase database), a central bulge with 4 nucleotide mismatches at positions 10 to 13 of the MBS and 4 nucleotide spacers between each MBS, according to Rama et al. [4]. This miR-21 sponge was inserted into pcDNA3.1 (+) CircRNA Mini Vector between two flanking sequences of ALU, in which the complementarity promoted the formation of a circle with the central sequence of the sponge during the back-splicing process [28], and as the reporter gene, the enhanced green fluorescence protein (EGFP) was inserted downstream of the CMV promoter and outside of the ALU sequences; therefore, its expression is proportional to the amount of Circ-21 sponge that is formed (~83% transfection efficiency). pcDNA3.1 (+) CircRNA Mini Vector has been used in recent studies to produce circular RNAs [40,41,42,43,44,45]. Zhang et al. [46,47] used this vector to create the circular RNA hsa_circ_0001445 and the hsa_circ_0001649 to regulate the proliferation, and the migration of hepatocellular carcinoma. Liang et al. [48] used the pcDNA3.1 (+) CircRNA mini vector for overexpression of circβ-catenin and to rescue the shRNA-mediated phenotypes in liver cancer cells. There are other circulating vectors that contain introns derived from an orthogonal gene, such as those of Zkscan1 [28], Hipk3 [28,49] and Laccase2 [50]. The HY_pMT vector uses 150 nucleotides of the native intronic sequence of the Laccase2 gene in Drosophila [51].

circRNA sponges are more stable than linear RNA sponges because they are covalently closed single-stranded RNAs that lack 5′ ends and 3′ poly-A structures, and are therefore inherently resistant to exonucleolytic RNA degradation [27,31,32,33]. Jeck et al. [26] identified more than 25,000 non-linear RNA species in human fibroblasts by enriching them with exonucleases. circRNAs were more stable than linear RNAs after being degraded by exonucleases, and the amount of circRNA was 10 times greater than the amount of linear RNA. Based on these characteristics, circRNA molecules may play an important role in the control of gene expression. circHIPK3 is a circRNA that functions as a circular sponge for miR-124 in hepatocellular carcinoma [52], and circ-ITCH is another circRNA that inhibits the progression of bladder cancer by acting as a sponge for miR-17 and miR-224 [53]. ciRS-7 is a naturally occurring endogenous circRNA expressed in the brain that is capable of inhibiting miR-7 activity. Hansen et al. [54] introduced CIRS-7 into zebrafish, observing a significant reduction in brain size, suggesting that CIRS-7 had a sponge function for miR-7. miR-122 is necessary for the life of the hepatitis C virus (HCV). Jost et al. [32] synthesized a circRNA capable of fluffing miR-122 in HuH-7.5 cells (human hepatoma-derived cell line) and thereby inhibiting HCV RNA translation. The inhibitory efficacy of circRNA was significant and comparable to the effect of the drug Miravirsen, which also exerts its inhibitory action on the virus through its complementary binding to miR-122.

Overexpression of miR-21 is related to the processes of cell proliferation, migration and apoptosis [55]. Circ-21 induced a significant and time-dependent decrease in A549 and LL2 cell growth, but low levels of growth inhibition were detected in L132 cells, which was similar to the results of those obtained by transfection with Circ-EGFP and untransfected parental lines. This reduction has also been observed in gastric carcinoma, where a Circ-21 sponge inhibited cell proliferation and suppressed the activity of miR-21 [56]. Our results also corroborate with trials using anti-miR-21, where a reduction in proliferation was detected in the A549 line cell [57] and other types of cancer [58]. Muller et al. [59] designed a synthetic circular RNA sponge containing four repetitive binding elements for miR-21, which reduced the proliferation of lung A549 cells and enhanced the expression of tumor suppressors (PDCD4, MOAP1, BTG1, BTG2, and FOXP1). Similar results were shown in A549 and H1975 miR-21 knockout cells, in which all five tumor suppressors showed higher levels of mRNA and protein. An in vivo study with A549-derived subcutaneous tumor xenograft model in mice revealed decreased tumor growth. Similarly, less migration was observed in the A549 line cell transfected with Circ-21 compared to that transfected with Circ-EGFP and without transfection. Yang et al. [60] detected an inhibition of the migration of the A549 cell line transfected with a miR-21-sponge at 24–72 h compared with no transfected cells. As in the previous assays, no decrease in migration area was detected in the non-tumor line L132. This test could not be performed with the LL2 line since, to perform it, a confluence of 90% was necessary to create the wound. This was not possible, since when trying to make the wound, the monolayer culture detached. In addition, the formation of colonies shown in A549 and LL2 cell lines transfected with Circ-21 was less than those transfected with Circ-EGFP and cells that were not transfected. This was not observed in the L132 cell line.

Based on these findings, we conducted a 3D multicellular tumor spheroid (MTS) study. Both A-549 and LL2 MTSs showed a significant decrease in volume growth after Circ-21 transfection, but no difference was recorded when transfected with Circ-EGFP or in the untransfected cell lines (control). In contrast, this behavior was not repeated for the non-tumor L132 line in which the growth of the MTS was similar for all of the experimental conditions. These results were corroborated with the antiproliferative effect of Circ-21 in lung cancer cells, previously observed in cell cultures.

Actually, the effect of the circ-21 sponge versus the controls is a good indication that Circ-21 is directly sponging miR-21. This interaction of miR-21 with their circ-21 could be confirmed by interaction analyses (immunoprecipitation or RNA pulldown-experiments).

## 4. Materials and Methods

### 4.1. Cell Culture

A549 human lung adenocarcinoma epithelial cell line, LL2 mouse Lewis lung carcinoma cell line and L132 human embryonic lung cell line were provided by the Instrumentation Service Center of the University of Granada, Granada, Spain. We chose A549 because it is one of the lung cancer lines cited in the literature with the highest levels of miR-21 expression. Line L132 was chosen as a control of healthy lung tissue in which miR-21 expression is low. and the LL2 line was chosen in case of future in vivo tests. All cell types were grown in Dulbecco’s Modified Eagle’s Medium (DMEM) (Sigma, St. Louis, MO, USA), supplemented with 10% fetal bovine serum (FBS) and 1% streptomycin-penicillin (Sigma), under air containing 5% CO_2_ and in an incubator at 37 °C.

### 4.2. miR-21 Expression Levels

RNA was extracted from the A549, LL2 and L132 cells with the miRCURY LNA miRNA PCR Starter Kit (Qiagen, Germantown, MD, USA) according to the manufacturer’s instructions. Then, the quantitative real time-polymerase chain reaction (qPCR) was performed using miRCURY SYBR Green PCR Master Mix (contained in the aforementioned kit) according to the manufacturer’s instructions. The thermocycling parameters were as follows: 95 °C for 2 min, followed by 40 cycles of 95 °C for 10 s, and 60 °C for 60 s. qPCR assays were performed on an ABI 7900 system (ABI), and the 2^−ΔΔCt^ method was applied for the calculation of relative levels of expression. hsa-miR-21-5p (YP00204230, Qiagen, Germantown, MD, USA)) was used to detect miR-21 expression levels, and has-miR-103-3p (YP00204063, Qiagen, Germantown, MD, USA) and has-miR-191-5p (YP00204306, Qiagen, Germantown, MD, USA) were used as the internal references.

### 4.3. Sponge Design and Construction

The hsa-miR-21-5p sequence was provided by the miRNABase (miRBase) database (https://www.mirbase.org/, accessed on 5 January 2022). The microRNA sponge sequence chosen based on optimizations previously was described by Rama et al. [4]. The features adopted here were based on Rama et al. [4]: 7 microRNA binding sites (MBS) [61,62,63]; a central bulge with 4 nucleotide mismatches at positions 10 to 13 of the MBS [63,64]; and 4 nucleotide spacers between each MBS [62,63,64]. For directional cloning within the region between both sequences Alu, the specific restriction site for *EcoRV* and *SacII* enzymes were added to the 5′ and 3′-ends, respectively. The sponge was synthesized (IDT, Coralville, IO, USA) and subcloned into pcDNA3.1 (+) CircRNA mini vector (Addgene, Teddington, UK). The enhanced green fluorescence protein (EGFP), which was used as a reporter gene, was cloned between *HindIII* and *EcoRI*. Subcloned bacterium for miR-21 sponge vector (Circ-21) were verified by DNA sequencing (data not shown).

### 4.4. Transfection of Cell Lines

The day before transfection, the A549, LL2 and L132 cell lines were seeded in their respective culture medium and incubated overnight. The transfection of Circ-21 and Circ-EGFP was performed with Lipofectamine 2000 (Invitrogen, Carlsbad, CA, USA), following the instructions of the manufacturer. The experiments were performed in the following groups: cells transfected with Circ-21, cells transfected with Circ-EGFP and control cells (no transfected cells).

### 4.5. Detection of Circ-21 Expression by RT-PCR In Vitro

Sponge expression was detected by RT-PCR. RNA was extracted from A549, LL2 and L132 cell lines (transfected with Cir-EGFP, Circ-21 and non-transfected) with the Rneasy Mini kit ((Qiagen, Germantown, MD, USA) 24 h after transfection. cDNA was generated by means of the Promega Reverse Transcription System (Promega, Madrid, Spain) using total cellular RNA (1 µg). Polymerase chain reaction (PCR) amplification of the EGFP-miR-21 sponge was performed to determine correct sponge expression as described above. Amplified PCR products were separated by 1.5% agarose gel electrophoresis and visualized with RedSafe Nucleic Acid Staining Solution (iNtRON Biotechnology, Korea, Japan). The images were scanned using a Bio-Rad documentation system (Quantity One Analysis Software, Bio-Rad, Hercules, CA, USA) (version 4.6.8).

### 4.6. Detection of Circ-21 Expression by Flow Cytometry

We used *EGFP* as a gene reporter to know the transfection efficiency. 24 h after transfection, all cell lines were collected, and the EGFP expression levels were analyzed using the Cellfit program with a FACScan flow cytometer (Becton Dickinson, San Jose, CA, USA).

### 4.7. Microscopic Analysis

*EGFP* was used as a reporter gene in the used constructions as described above. EGFP was excited at 488 nm and fluorescence microscopy analysis was conducted with a Leica DMI6000 microscope (Heidelberg, Germany).

### 4.8. In Vitro Cell Proliferation Assay

The A549, LL2 and L132 cell lines were seeded in 96-well plates (7 × 10^3^ cells/well) and transfected as described above. The experiments were performed in the following groups: cells transfected with Circ-21, cells transfected with Circ-EGFP and control cells (no transfected cells). MTT (3-(4,5 dimethylthiazol-2-yl)-2,5-diphenyltetrazolium bromide) solution (5 mg/mL) was added to each well (10 μL) and incubated for 4 h at 37 °C. One hundred microliters of dimethylsulfoxide (DMSO) were then added to each well after the medium had been removed. Optical density was determined using a Titertek multiscan colorimeter (Flow Laboratories, Oldham, UK) at 570 and 690 nm. The proliferation effect of miR-21 was determined at 24, 48 and 72 h.

### 4.9. Wound Healing Assay

To determine the tumor cell migration capacity of cell lines and, therefore, their invasiveness and ability to generate metastases, an in vitro migration assay was performed. The A549, LL2 and L132 cell lines were seeded in 24-well plates (15 × 10^4^ cells/well) and transfected as described above. Upon reaching 90% confluence, a “wound” was created using a sterile 100 pipette tip following Grada et al. [65] Then, two washes were performed using 500 µL of PBS. Finally, 800 µL of DMEM without FBS was added to each well. The monitoring of cell migration was performed by taking images at times 0, 24, 48 and 72 h using a Leica microscope (Wetzlar, Germany). The results were analyzed using MRI Wound Healing Tool of ImageJ software (version 1.52s) (National Institutes of Health, Bethesda, MD, USA, https://imagej.nih.gov/ij/, accessed on 5 January 2022).

### 4.10. Colony Formation

The A549, LL2 and L132 cell lines were seeded in 12-well plates (600 cells/well) and transfected as described above. One week after seeding, the wells were washed twice using 1 mL of PBS, and the cells were fixed using 1 mL/well cold 70% methanol for 30 min. Then, the cells were washed twice with water, and the plates were dried overnight. Colonies were stained using 1 mL/well 0.5% crystal violet in 70% methanol, and the plates were incubated for 15 min on an orbital shaker. Afterward, plates were washed three times using water and were dried overnight. Finally, colony number was counted. All experiments were performed in triplicate.

### 4.11. Multicellular Tumor Spheroids Generation

The A-549, LL2 and L132 cells (5 × 10^2^ cells/well) were grown in a 96-well microplate (BD Biosciences, Madrid, Spain) to generate multicellular tumor spheroid (MTS) according to Rama et al. [66,67]. Once the cells were seeded, the plates were centrifuged at 500× *g* for 10 min to promote aggregation. MTSs were formed after three days. They were grown in Dulbecco’s Modified Eagle’s Medium (DMEM) (Sigma, St. Louis, MO, USA), supplemented with 10% fetal bovine serum (FBS) and 1% streptomycin-penicillin (Sigma), under air containing 5% CO_2_ and in an incubator at 37 °C.

### 4.12. MTS Growth Volume Assay

MTSs were treated in the same groups and in a similar manner as those described for cell cultures (mentioned above). Growth of the spheroids was monitored and measured to obtain a median relative volume (volume at day x/volume at day 0). Their volumes (V, mm^3^) were estimated by measuring the largest diameter “a” and the second largest diameter “b” perpendicular to “a”, and then by calculating the volume from V = a × b^2^ × π/6. All experiments were performed in quintuplicate.

### 4.13. Statistical Analysis

All the results are expressed as the means ± the standard deviation (SD). Statistical analysis was performed using Student’s *t*-test. All the tests were performed using the Statistical Package for the Social Sciences (SPSS) v. 15.0 with a significance level of 0.05 (α = 0.05).

## 5. Conclusions

We demonstrated that Circ-21 is an excellent sponge capable of inhibiting cell growth, decreasing migration and decreasing MTS volume. In contrast, normal lung cells were not damaged. These results suggest that circular sponges may be a tool for miRNA inhibition and that, in particular, Circ-21 may be an excellent candidate for treatment in this type of tumor. Interaction of miR-21 with their RNA sponge could be confirmed in future studies via interaction analyses (immunoprecipitation or RNA pulldown-experiments).

## Supplementary Materials

The following supporting information can be downloaded at: https://www.mdpi.com/article/10.3390/ijms23062963/s1.
