# Supplementary material for: Synthetic Circular miR-21 Sponge as Tool for Lung Cancer Treatment
Source: Int J Mol Sci. 2022 Mar 9;23(6):2963. doi: 10.3390/ijms23062963 (PMC8955967; doi:10.3390/ijms23062963)
Supplement: Supplementary file 1 [file ijms-23-02963-s001.zip › ijms-1587759-supplementary/Table S1.pdf]

Table S1: miRNA involved in lung cancer.

miRNA	Function	Reference
**Clúster miR-17-92**	Oncogenic	[1]
**miR-96**	Oncogenic	[2]
**miR-183**	Oncogenic	[2]
**miR-182**	Oncogenic	[2]
**miR-143**	Oncogenic	[3]
**miR-145**	Oncogenic	[3]
**miR-494**	Oncogenic	[4]
**miR-155**	Oncogenic	[5]
**let-7**	Tumor suppressor	[6]
**miR-126**	Tumor suppressor	[7]
**miR-34**	Tumor suppressor	[8]

References

1. Li, L.; Song, W.; Yan, X.; Li, A.; Zhang, X.; Li, W.; Wen, X.; Zhou, L.; Yu, D.; Hu, J.F.; et al. Friend leukemia virus integration 1 promotes tumorigenesis of small cell lung cancer cells by activating the miR-17-92 pathway. *Oncotarget* **2017**, *8*, 41975-41987, doi:10.18632/oncotarget.16715.

2. Dambal, S.; Shah, M.; Mihelich, B.; Nonn, L. The microRNA-183 cluster: the family that plays together stays together. *Nucleic acids research* **2015**, *43*, 7173-7188.

3. Skjefstad, K.; Johannessen, C.; Grindstad, T.; Kilvaer, T.; Paulsen, E.-E.; Pedersen, M.; Donnem, T.; Andersen, S.; Bremnes, R.; Richardsen, E. A gender specific improved survival related to stromal miR-143 and miR-145 expression in non-small cell lung cancer. *Scientific reports* **2018**, *8*, 8549.

4. Wang, J.; Chen, H.; Liao, Y.; Chen, N.; Liu, T.; Zhang, H.; Zhang, H. Expression and clinical evidence of miR-494 and PTEN in non-small cell lung cancer. *Tumor Biology* **2015**, *36*, 6965-6972.

5. Liu, M.; Zhou, K.; Huang, Y.; Cao, Y. The candidate oncogene (MCRS1) promotes the growth of human lung cancer cells via the miR–155–Rb1 pathway. *Journal of Experimental & Clinical Cancer Research* **2015**, *34*, 121.

6. Yang, G.; Zhang, W.; Yu, C.; Ren, J.; An, Z. MicroRNA let-7: Regulation, single nucleotide polymorphism, and therapy in lung cancer. *Journal of cancer research and therapeutics* **2015**, *11*, 1.

7. Rusek, A.M.; Abba, M.; Eljaszewicz, A.; Moniuszko, M.; Niklinski, J.; Allgayer, H. MicroRNA modulators of epigenetic regulation, the tumor microenvironment and the immune system in lung cancer. *Molecular cancer* **2015**, *14*, 34.

8. Grimolizzi, F.; Monaco, F.; Leoni, F.; Bracci, M.; Staffolani, S.; Bersaglieri, C.; Gaetani, S.; Valentino, M.; Amati, M.; Rubini, C. Exosomal miR-126 as a circulating biomarker in non-small-cell lung cancer regulating cancer progression. *Scientific reports* **2017**, *7*, 15277.
