# Supplementary material for: Synthetic Circular miR-21 Sponge as Tool for Lung Cancer Treatment
Source: Int J Mol Sci. 2022 Mar 9;23(6):2963. doi: 10.3390/ijms23062963 (PMC8955967; doi:10.3390/ijms23062963)
Supplement: Supplementary file 1 [file ijms-23-02963-s001.zip › ijms-1587759-supplementary/Table S2.pdf]

Table S2: Cancer types in which miR-21 is overexpressed.

Type of cancer	Reference
**Not small cells lung carcinoma**	[1]
**Small cells lung carcinoma**	[2]
**Glioblastoma**	[3]
**Breast cancer**	[4]
**Pancreatic cnacer**	[5]
**Gastric cancer**	[6]
**Chronic lymphocytic leukemia**	[7]
**Acute myeloid lymphoma**	[8]
**Myeloma**	[9]
**Hepatocellular carcinoma**	[10]
**Diffuse large B-cell lymphoma**	[11]
**Colorectal cancer**	[12]
**Cervical cancer**	[13]
**Esophageal cancer**	[14]
**Tongue squamous cell carcinoma**	[15]

**Bladder cancer**	[16]
**Ovarian cancer**	[17]
**Laryngeal cancer**	[18]

References

1. Markou, A.; Zavridou, M.; Lianidou, E.S. miRNA-21 as a novel therapeutic target in lung cancer. *Lung Cancer: Targets and Therapy* **2016**, *7*, 19.

2. Inamura, K.; Ishikawa, Y. MicroRNA in lung cancer: novel biomarkers and potential tools for treatment. *Journal of clinical medicine* **2016**, *5*, 36.

3. Masoudi, M.S.; Mehrabian, E.; Mirzaei, H. MiR-21: A key player in glioblastoma pathogenesis. *Journal of cellular biochemistry* **2018**, *119*, 1285-1290.

4. Fang, H.; Xie, J.; Zhang, M.; Zhao, Z.; Wan, Y.; Yao, Y. miRNA-21 promotes proliferation and invasion of triple-negative breast cancer cells through targeting PTEN. *American journal of translational research* **2017**, *9*, 953.

5. Abue, M.; Yokoyama, M.; Shibuya, R.; Tamai, K.; Yamaguchi, K.; Sato, I.; Tanaka, N.; Hamada, S.; Shimosegawa, T.; Sugamura, K. Circulating miR-483-3p and miR-21 is highly expressed in plasma of pancreatic cancer. *International journal of oncology* **2015**, *46*, 539-547.

6. Simonian, M.; Mosallayi, M.; Mirzaei, H. Circulating miR-21 as novel biomarker in gastric cancer: diagnostic and prognostic biomarker. *Journal of cancer research and therapeutics* **2018**, *14*.

7. Mirzaei, H.; Fathullahzadeh, S.; Khanmohammadi, R.; Darijani, M.; Momeni, F.; Masoudifar, A.; Goodarzi, M.; Mardanshah, O.; Stenvang, J.; Jaafari, M.R. State of the art in microRNA as diagnostic and therapeutic biomarkers in chronic lymphocytic leukemia. *Journal of cellular physiology* **2018**, *233*, 888-900.

8. Bolouri, H.; Farrar, J.E.; Triche Jr, T.; Ries, R.E.; Lim, E.L.; Alonzo, T.A.; Ma, Y.; Moore, R.; Mungall, A.J.; Marra, M.A. The molecular landscape of pediatric acute myeloid leukemia reveals recurrent structural alterations and age-specific mutational interactions. *Nature medicine* **2018**, *24*, 103.

9. Calura, E.; Bisognin, A.; Manzoni, M.; Todoerti, K.; Taiana, E.; Sales, G.; Morgan, G.J.; Tonon, G.; Amodio, N.; Tassone, P. Disentangling the microRNA regulatory milieu in multiple myeloma: integrative genomics analysis outlines mixed miRNA-TF circuits and pathway-derived networks modulated in t (4; 14) patients. *Oncotarget* **2016**, *7*, 2367.

10. Wagenaar, T.R.; Zabludoff, S.; Ahn, S.-M.; Allerson, C.; Arlt, H.; Baffa, R.; Cao, H.; Davis, S.; Garcia-Echeverria, C.; Gaur, R. Anti–miR-21 suppresses hepatocellular carcinoma growth via broad transcriptional network deregulation. *Molecular Cancer Research* **2015**, *13*, 1009-1021.

11. Musilova, K.; Mraz, M. MicroRNAs in B-cell lymphomas: how a complex biology gets more complex. *Leukemia* **2015**, *29*, 1004.

12. Wu, Y.; Song, Y.; Xiong, Y.; Wang, X.; Xu, K.; Han, B.; Bai, Y.; Li, L.; Zhang, Y.; Zhou, L. MicroRNA-21 (Mir-21) promotes cell growth and invasion by repressing tumor suppressor PTEN in colorectal cancer. *Cellular Physiology and Biochemistry* **2017**, *43*, 945-958.

13. Zhang, J.; Yao, T.; Wang, Y.; Yu, J.; Liu, Y.; Lin, Z. Long noncoding RNA MEG3 is downregulated in cervical cancer and affects cell proliferation and apoptosis by regulating miR-21. *Cancer biology & therapy* **2016**, *17*, 104-113.

14. Liao, J.; Liu, R.; Shi, Y.-J.; Yin, L.-H.; Pu, Y.-P. Exosome-shuttling microRNA-21 promotes cell migration and invasion-targeting PDCD4 in esophageal cancer. *International journal of oncology* **2016**, *48*, 2567-2579.

15. Li, L.; Li, C.; Wang, S.; Wang, Z.; Jiang, J.; Wang, W.; Li, X.; Chen, J.; Liu, K.; Li, C. Exosomes derived from hypoxic oral squamous cell carcinoma cells deliver miR-21 to normoxic cells to elicit a prometastatic phenotype. *Cancer research* **2016**, *76*, 1770-1780.

16. Yang, X.; Cheng, Y.; Li, P.; Tao, J.; Deng, X.; Zhang, X.; Gu, M.; Lu, Q.; Yin, C. A lentiviral sponge for miRNA-21 diminishes aerobic glycolysis in bladder cancer T24 cells via the PTEN/PI3K/AKT/mTOR axis. *Tumor Biology* **2015**, *36*, 383-391.

17. Báez-Vega, P.M.; Vargas, I.M.E.; Valiyeva, F.; Encarnación-Rosado, J.; Roman, A.; Flores, J.; Marcos-Martínez, M.J.; Vivas-Mejía, P.E. Targeting miR-21-3p inhibits proliferation and invasion of ovarian cancer cells. *Oncotarget* **2016**, *7*, 36321.

18. Yu, X.; Li, Z. The role of microRNAs expression in laryngeal cancer. *Oncotarget* **2015**, *6*, 23297.
